# Dynamics of longitudinal growth in children with idiopathic rheumatoid arthritis in response to infliximab treatment

**DOI:** 10.1186/1546-0096-9-S1-P165

**Published:** 2011-09-14

**Authors:** OU Konopelko, ES Zholobova, OS Rozvadovskaya

**Affiliations:** 1 Department of Pediatric Rheumatology, Leuven, Belgium, First Moscow Medical State University I.M. Sechenov, Moscow, Russian Federation

## Background

Anti-tumor necrosis factor (TNF) – infliximab is known to decrease disease activity of juvenile idiopathic arthritis (JIA) but its effect on longitudinal growth in relation to puberty is not clear.

## Aim

To assess the dynamics of longitudinal growth in prepubertal and pubertal patient’s with JIA in response to infliximab, to evaluate effectiveness of the therapy.

## Materials and methods

Study included 13 children, 8 of the prepubertal and 5 - the puberty. The study was subjected to information about sex, age, children’s growth, diagnosis, therapy. Growth was estimated by measuring and comparing patient’s height standard deviation score(SDS) in relation to the midparental height, the change of this value (ΔhSDS) from -1 to 0 and 0 to 1 year of treatment and the change between the ΔhSDS values to assess growth improvement. Treatment efficacy was assessed according to criteria ACR pedi. Infliximab was administered in connection with ineffectiveness of standard antirheumatic therapy (methotrexate, leflunamide, cyclosporine A, methotrexate + cyclosporine A combination).

## Results

9 of all 13 children (69,2%) were with systemic JIA, 2 of all 13 children (15,4%) – poliarticular JIA, other 2 of 13 children (15,4%) – juvenile spondyloarthritis. Before infliximab theatment all children were with 3-2 degrees of disease activity. After a year of infliximab 53.8% of patients demonstrated response ACR pedi70, 30,8% of patients - ACR pedi 50, 15,4% - ACR pedi 30. In the prepubertal group the relation height SDS (mean ± standart error of the mean) was -1.53 ± 1,-2.07 ± 1 and 1.45 ±1.61 at -1, 0 and 1 year of infliximab treatment respectively. The ΔhSDS before infliximab was -0.54 ± 0.27, over the first year with infliximab ΔhSDS was 0.62 ± 0.9(p>0,05). In the pubertal group the SDS was-1, 39 ± 2.36, -3.12 ± 2.6, and -3.12 ± 2.61 at -1, 0 and 1 year of treatment respectively. The ΔhSDS before infliximab was -0.73 ± 0.43, over the first year with infliximab ΔhSDS was -0.004 ± 0.47 (p>0,05). Individual analysis of each patient revealed that most children of the pubertal group (3 / 5) and the prepubertal group (6 / 8) showed improvement in longitudinal growth during treatment with infliximab. Out of the 13 children included in the study prior to the infliximab, 8 received steroids per os. After a year of the treatment in therapy of 6 children doses of steroids were decreased and one child no longer needed steroids. The dose of steroids mg / kg / day to 0 and 1 year of the therapy respectively was 0.22 -0.1, 0.12 -0.1, 0.1 -0.076 Δdozy.

## Conclusions

Infliximab treatment in addition to a significant therapeutic effect also showed an increase of longitudinal growth in children suffering from idiopathic rheumatoid arthritis.

